# National Dental Hospital

**Published:** 1861-09

**Authors:** 


NATIONAL DENTAL HOSPITAL.—A national dental hospital was recently established in the British metropolis, for the benefit of the poor suffering from diseases of the teeth, gums, and jaws. Dr. Pavy, of Guy's Hospital, and Dr. Richardson, were appointed consulting physicians; Mr. Erichsen and Mr. Spencer Wells, consulting surgeons; and Messrs. Wait, Merryweather, and Robinson, consulting dentists.

